# Comparing visual outcomes in matched patients receiving a low cylinder power toric IOL or a non-toric IOL

**DOI:** 10.3389/fopht.2026.1755069

**Published:** 2026-07-23

**Authors:** Kjell G. Gundersen, Richard Potvin, Steffen A. Østenstad

**Affiliations:** 1 IFocus Øyeklinikk AS, Haugesund, Norway; 2 Science in Vision, Frisco, TX, United States

**Keywords:** astigmatism, cataract, Clareon, low cylinder, toric IOL

## Abstract

**Purpose:**

To evaluate visual and refractive outcomes of the Alcon Clareon^®^ T2 toric IOL (1.00 D cylinder at the IOL plane, ~ 0.65 D at the corneal plane) versus the Clareon^®^ non-toric IOL.

**Setting:**

Single ophthalmology clinic in Haugesund, Norway.

**Methods:**

This was an exploratory single site randomized, unmasked, prospective clinical trial. The study was registered with clinicaltrials.gov (NCT06717607, registered retrospectively on 12/5/2024). Patients presenting for routine cataract surgery in one or both eyes, where at least one eye qualified for implantation of the T2 lens, were enrolled. Qualifying eyes were randomized to a T2 lens or a non-toric lens. Subjects were evaluated monocularly before surgery and three months postoperative. Testing included uncorrected and corrected distance visual acuity (VA), the manifest refraction, low contrast VA (photopic and mesopic, with and without glare) and contrast sensitivity (photopic and mesopic). These latter two tests were conducted with the distance correction in place.

**Results:**

Fifty eyes of 43 subjects were successfully enrolled from 6/2022 to 5/2024, including 30 eyes (25 subjects) randomized to the toric IOL and 20 eyes (18 subjects) to the non-toric IOL. Analysis included all enrolled eyes. Subjects were matched for age (p = 0.59), sex (p = 0.30), mean keratometry (p = 0.44), anterior corneal astigmatism (p = 0.97), axial length (p = 0.12) and anterior chamber depth (p = 0.40). Postoperatively, there was no statistically significant difference in the spherical equivalent refraction (p = 0.08). Residual refractive cylinder was statistically significantly lower in the T2 group (0.22 ± 0.22 D vs. 0.59 ± 0.28 D, p < 0.01). The T2 group also had statistically significantly better uncorrected logMAR distance VA than the non-toric group (0.00 ± 0.08 vs. 0.09 ± 0.13, p < 0.01). Low contrast VA was not statistically significantly different between the two lens types under any test condition (p = 0.49 or higher in all cases), nor were photopic or mesopic contrast sensitivity (p = 0.71 and p = 0.63, respectively).

**Conclusions:**

Use of the low cylinder power toric IOL provided significantly better UDVA, with no significant differences in distance-corrected contrast sensitivity or low contrast acuity. The achieved cylinder correction was consistent with expectations.

**Clinical trial registration:**

https://clinicaltrials.gov/, identifier NCT06717607.

## Introduction

For more than 25 years, toric intraocular lenses (IOLs) have been widely available to patients presenting for cataract surgery. Numerous studies have demonstrated the effectiveness of these lenses. Compared to non-toric IOLs (with or without concurrent relaxing incisions), evidence indicates that they provide better uncorrected distance visual acuity (UDVA), lower levels of residual refractive astigmatism and greater spectacle independence (1).

The clinical performance of toric IOLs depends on several factors. Preoperatively, these include accurate biometry and compensation for posterior corneal astigmatism (PCA) and surgically induced astigmatism in the toric IOL calculation. At the time of surgery, accurate orientation of the lens is required. Postoperatively, rotational stability is a critical factor (2). Another variable is the lens position after surgery (postoperative anterior chamber depth), which will affect the actual cylinder power of the IOL at the cornea (the cylinder power of the IOL is known only at the IOL plane) (3). As the cylinder power of the lens decreases, the variability in these factors can become a significant limitation on achieving the intended correction. Improvements in biometry, new toric IOL calculation formulas that compensate for PCA, and better systems for intraoperative toric IOL orientation have made it possible to consider correcting lower levels of astigmatism at the time of cataract surgery.

Accurate correction of low amounts of anterior corneal astigmatism is of interest because prevalence data suggests 45% of cataractous eyes have anterior corneal astigmatism from 0.50 to 1.00 D (4). Many of these eyes would likely benefit from correction with a toric IOL (5). While corneal arcuate incisions can be used to correct this magnitude of corneal astigmatism, evidence suggests that toric IOLs provide more predictable and less variable outcomes (6, 7). In addition, evidence shows that even small amounts of uncorrected astigmatism can negatively affect vision; the effects on functional vision appear higher than the effects on high contrast visual acuity (VA) (8). Several other potentially confounding factors in evaluating the correction of low amounts of cylinder are the test-retest reliability of refractions (9), and the small changes in refraction that can occur with pupil size changes (2), though the effects from these are likely to be minor.

A relatively new IOL is the Clareon^®^ lens (CNW0TT, Alcon, Fort Worth, USA). It is made from a new hydrophobic acrylic material, with a 6 mm aspheric optic and C-loop haptics. The toric IOL design is similar to that of the predecessor SN6ATT lens. The lowest cylinder power (T2) of this IOL is 1.0 D at the IOL plane, corresponding to about 0.65 D at the corneal plane. The rotational stability of the lens has been demonstrated, and clinical results for the T3 to T9 versions of the lens have been reported (10).

The purpose of the current study was to compare visual and refractive outcomes between the low toric IOL (Clareon T2) and the non-toric Clareon IOL (NT).

## Patients and methods

This was an exploratory single-site randomized, unmasked prospective clinical trial. Approval for the study was obtained from the local ethics committee (REK, Regionale komiteer for medisinsk og helsefaglig forskningsetikk, Norway, approval number 370677, 16/5/2022). All subjects signed an informed consent document. The study adhered to the tenets of the Declaration of Helsinki and Good Clinical Practice. The study was registered with clinicaltrials.gov (NCT06717607, registered retrospectively on 12/5/2024). CONSORT guidelines for clinical trials were followed.

At the enrollment visit all subjects were qualified for the study. Potential subjects had to be patients presenting for cataract surgery, more than 40 years old, with good ocular health and no pathology likely to compromise postoperative visual acuity (VA). Potential postoperative VA had to be 0.2 logMAR (20/32) or better. Exclusion criteria included previous ocular surgery (corneal, anterior or posterior), diabetic retinopathy, and any acute or chronic disease (e.g. atopy, immunocompromise) deemed likely to confound results of the study. Biometry was performed using the LS 900 biometer (Haag-Streit, Kõniz, Switzerland) with the IOL toric power determined using the Barrett Toric calculator, with consideration of posterior corneal astigmatism, as well as the expected surgically induced astigmatism from the planned incision. Any eye with a T2 IOL recommended was eligible for enrollment.

Biometry was repeated on the day of surgery to confirm eligibility. If eligible, the eye was randomly assigned to the T2 or the non-toric (NT) IOL using a pre-generated list prepared using an Excel random number generator (Microsoft, Redmond, USA). If both eyes of a subject were eligible for inclusion, then the same lens (T2 or NT) was implanted in both eyes. Surgery was performed on all subjects by a single surgeon (KGG) using their usual standard of care, with a 2.2 mm superior incision for all cases. For eyes implanted with the T2 IOL, the Verion™ Image Analysis System (Alcon, Fort Worth, TX) was used for intraoperative alignment of the toric axis. Subjects were seen one day and 3 months after bilateral surgery. At the one day and 3 month visits the orientation of the IOL was determined, using a slit lamp. Postoperative treatment and the medication regimen were standard for the practice. All visits included monitoring for adverse events.

At the 3-month visit, the manifest refraction for each eye was determined. All visual acuity and contrast sensitivity testing was performed using the Clinical Trial Suite (CTS) from M&S Technologies (Niles, IL, USA), following the instructions for use for each of the relevant tests. Monocular VA testing was conducted under photopic lighting conditions, both uncorrected and with the distance correction in place. Low contrast (10%) VA was measured in both photopic and mesopic conditions, with and without glare, at 4 m. Contrast sensitivity was measured using sine wave gratings, in both photopic and mesopic conditions. Both contrast tests were conducted monocularly, with the distance correction in place.

The primary outcome measure of interest was the uncorrected distance visual acuity (UDVA) at 4m. Secondary outcome measures included the residual refraction, the corrected distance visual acuity (CDVA), low contrast VA in photopic and mesopic conditions, with and without glare, and contrast sensitivity. Assuming a standard deviation in UDVA of 0.1 logMAR units, with an alpha of 0.05 and a power of 0.8, 17 eyes would be required to reliably detect a 1 logMAR line (0.1) difference in UDVA. Target enrollment was up to 25 eyes in each group, to allow for possible dropout, but patient availability was a limiting factor.

Data were exported from the Clinical Trial System and collated in Microsoft Excel, then imported into Microsoft Access for review and data checking (both Microsoft Corp., Redmond, USA). Statistica version 13 was used for the statistical analyses (Tibco Software, Palo Alto, USA). Parametric variables were compared using analysis of variance (ANOVA), while categorical data were analyzed using the Chi-squared test. In all cases, statistical significance was set to (p ≤ 0.05).

## Results

**Figure 1:**
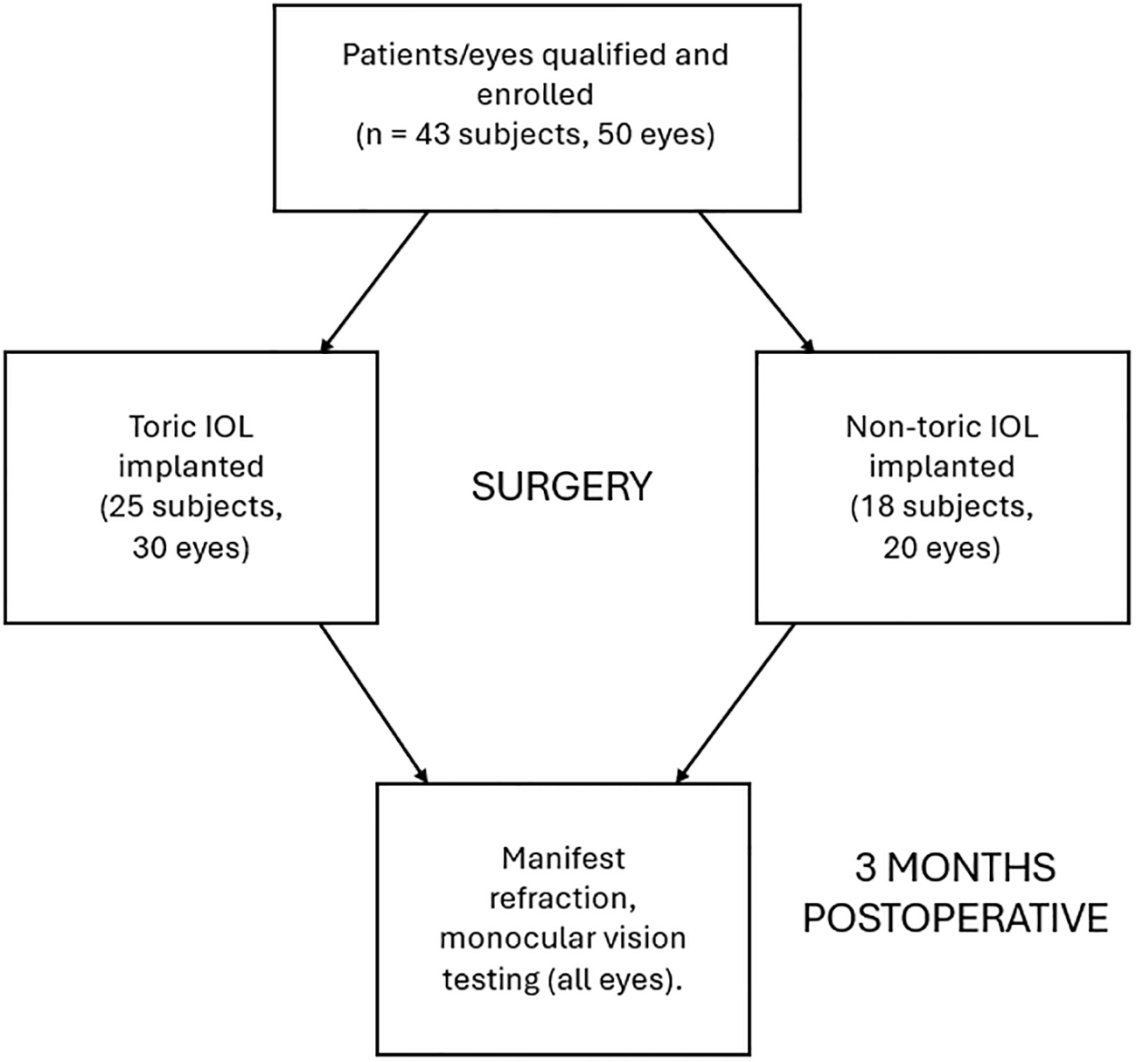
Patient flow through the study.

Fifty eyes of 43 subjects were successfully enrolled from 6/2022 to 5/2024. **Figure 1** shows the flowchart of patients and eyes through the study. No patients were lost to follow up and no subjects required observation past the 3-month visit. All analyses were monocular, and all eyes were included (30 T2, 20 non-toric).

**Table 1** shows the subject characteristics, and eye characteristics for the two groups. Analysis showed that subjects were matched for age, sex, mean keratometry, anterior corneal astigmatism, axial length and anterior chamber depth. There was no statistically significant difference in the preoperative refractive cylinder or the distance-corrected visual acuity (DCVA). The preoperative mean refraction spherical equivalent (MRSE) was statistically significantly different (slightly more myopic in the NT group), but the difference was not considered clinically important.

**Table 1:** Demographic and preoperative data summary.

Variables	T2	Non-toric	P
Subjects/eyes	25/30	18/20	
Age	74 ± 6 (60 to 85)	73 ± 7 (59 to 84)	0.55
Sex (M/F)	12/13	7/11	0.55
Mean keratometry (D)	43.72 ± 1.60 (39.75 to 47.48)	43.36 ± 1.52 (41.00 to 46.09)	0.44
Anterior corneal astigmatism (D)	0.84 ± 0.47 (0.25 to 1.55)	0.84 ± 0.36 (0.29 to 1.37)	0.97
Axial length (mm)	23.58 ± 0.86 (21.42 to 25.27)	23.96 ± 0.78 (22.47 to 25.60)	0.12
Anterior chamber depth (mm)	3.11 ± 0.36 (2.46 to 3.75)	3.20 ± 0.0.35 (2.66 to 3.90)	0.40
Preoperative MRSE (D)	0.24 ± 1.77 (-3.50 to 3.25)	-1.17 ± 2.13 (-5.75 to 2.00)	0.014
Preoperative refractive cylinder (D)	0.94 ± 0.52 (0.25 to 2.50)	0.96 ± 0.50 (0.25 to 2.00)	0.89
Preoperative DCVA (logMAR)	0.21 ± 0.13 (0.02 to 0.62)	0.19 ± 0.13 (0.00 to 0.40)	0.59

MRSE, mean refraction spherical equivalent; D, diopter; DCVA, distance corrected visual acuity.

Values are mean ± standard deviation (range).

The toric calculator for all eyes factored in posterior corneal astigmatism and the surgeon’s surgically induced astigmatism to calculate the predicted postoperative residual refractive astigmatism. The results showed a statistically significantly lower predicted residual refractive astigmatism magnitude with the T2 lens, as expected. The data are summarized in **Table 2**. The difference in the mean predicted residual refractive astigmatism between groups was 0.60 D, very close to the nominal power of the T2 IOL at the corneal plane.

**Table 2:** Predicted and actual refractive and visual acuity results.

Variables	T2	Non-toric	P
Predicted refractive astigmatism magnitude (D)	0.08 ± 0.06 (0.00 to 0.19)	0.68 ± 0.26 (0.11 to 1.28)	< 0.01
Postoperative MRSE (D)	0.22 ± 0.42 (-0.38 to 1.13)	0.47 ± 0.55 (-0.50 to 1.50)	0.08
% of eyes with MRSE within 0.50 D of plano	80%	55%	0.06
Postoperative refractive cylinder (D)	0.23 ± 0.22 (0.00 to 0.75)	0.59 ± 0.28 (0.00 to 1.50)	< 0.01
% of eyes with refractive cylinder ≤ 0.25 D	73%	20%	< 0.01
% of eyes with refractive cylinder ≤ 0.50 D	97%	55%	< 0.01
UDVA (logMAR)	0.00 ± 0.08 (-0.18 to 0.24)	0.09 ± 0.13 (-0.10 to 0.30)	< 0.01
% of eyes with UDVA 0.1 logMAR (20/25) or better	93%	65%	0.01
CDVA (logMAR)	-0.07 ± 0.05 (-0.18 to 0.04)	-0.04 ± 0.07 (-0.14 to 0.08)	0.11

MRSE, mean refraction spherical equivalent; D, diopter; UDVA, uncorrected distance visual acuity; DCVA, distance corrected visual acuity.

Values are mean ± standard deviation (range).

Also included in **Table 2** are the postoperative MRSE and the magnitude of the postoperative refractive cylinder. There was no statistically significant difference in the MRSE between groups. Eighty percent of eyes in the T2 group and 55 percent of eyes in the NT group were within 0.50 D of plano, with a tendency for the eyes in the NT group to be slightly more hyperopic.

Postoperative refractive cylinder was statistically significantly lower in the T2 group. The mean difference between the two groups was 0.36 D. The percentages of eyes with ≤ 0.25 D and ≤ 0.50 D of refractive cylinder were statistically significantly higher in the T2 group (see **Table 2**).

The T2 group also had statistically significantly better uncorrected distance visual acuity (UDVA) than the NT group, with almost a line better acuity. Significantly more eyes in the T2 group had a UDVA of 0.1 logMAR (20/25) or better. The corrected distance visual acuity (CDVA) was not statistically significantly different between the groups (**Table 2**).

**Figure 2:**
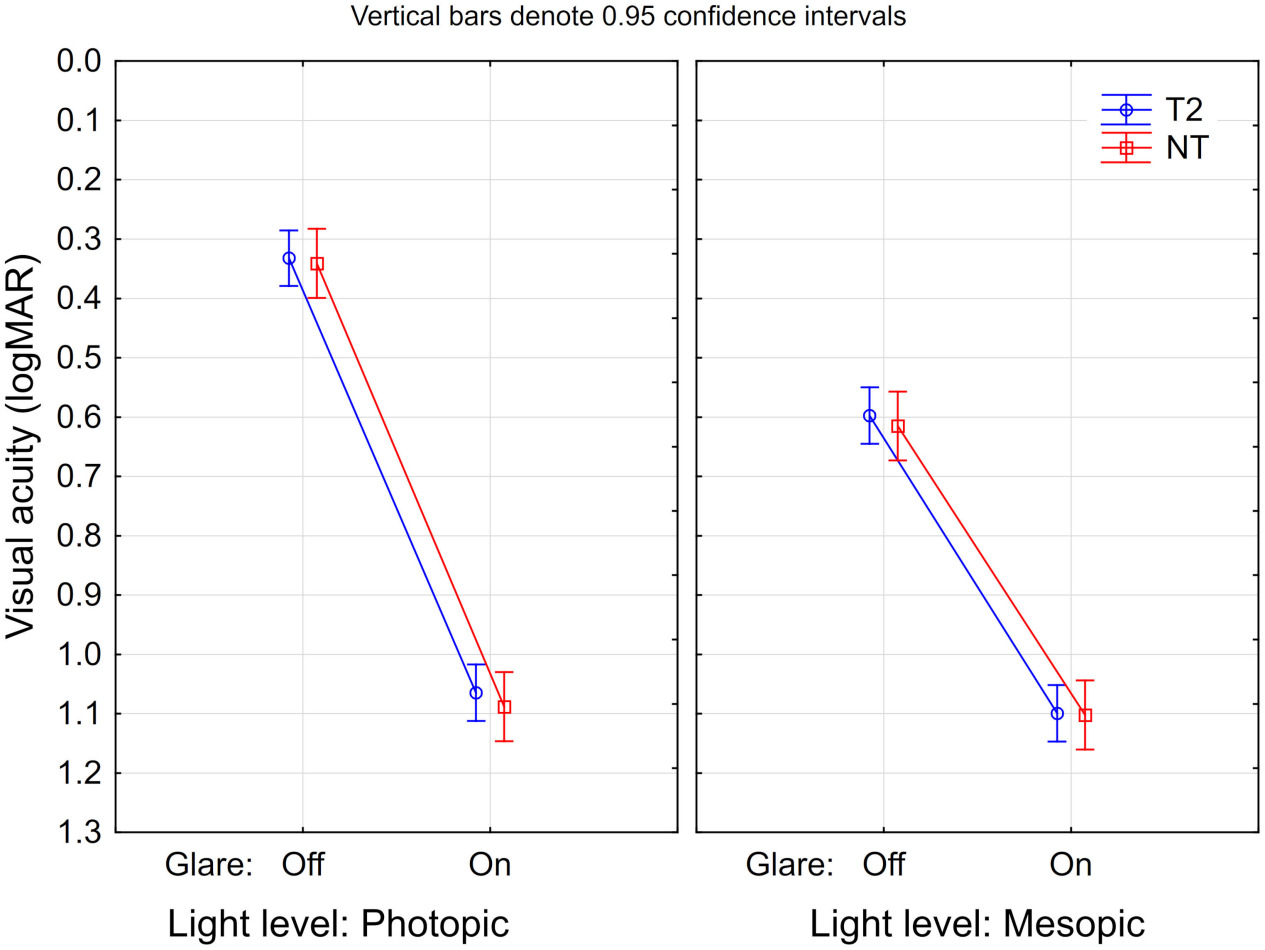
Low contrast visual acuity by lighting, glare and lens type.

**Figure 2** shows the results of the low contrast VA testing. As expected, VA was reduced relative to high contrast VA, worse in the mesopic test condition, and negatively affected by glare in both photopic and mesopic conditions. However, there were no statistically significant differences between the two lens types under any of the test conditions (p = 0.49). As noted in the methods, the distance correction was in place for these tests.

**Figure 3:**
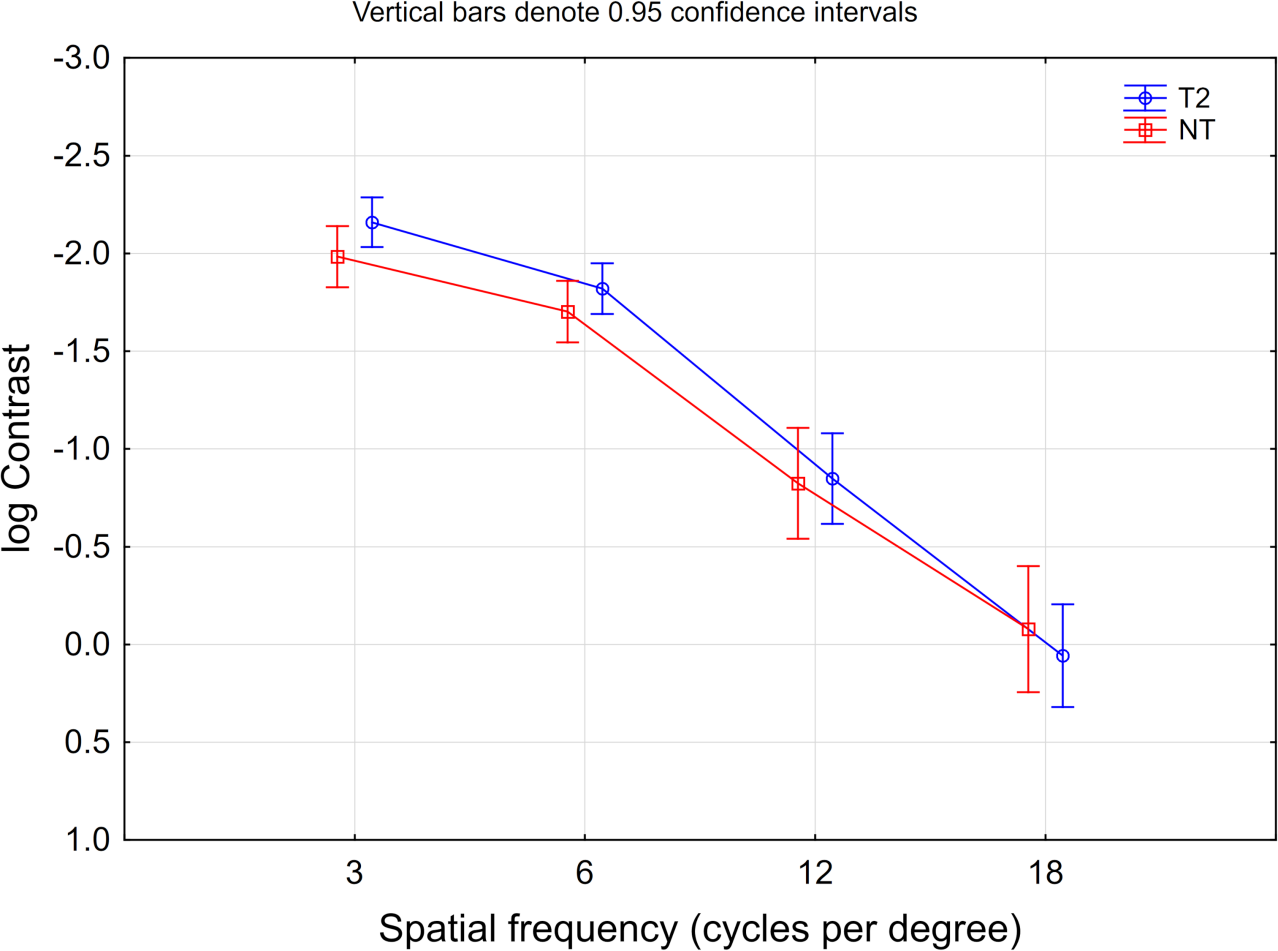
Photopic contrast sensitivity by lens type.

**Figure 4:**
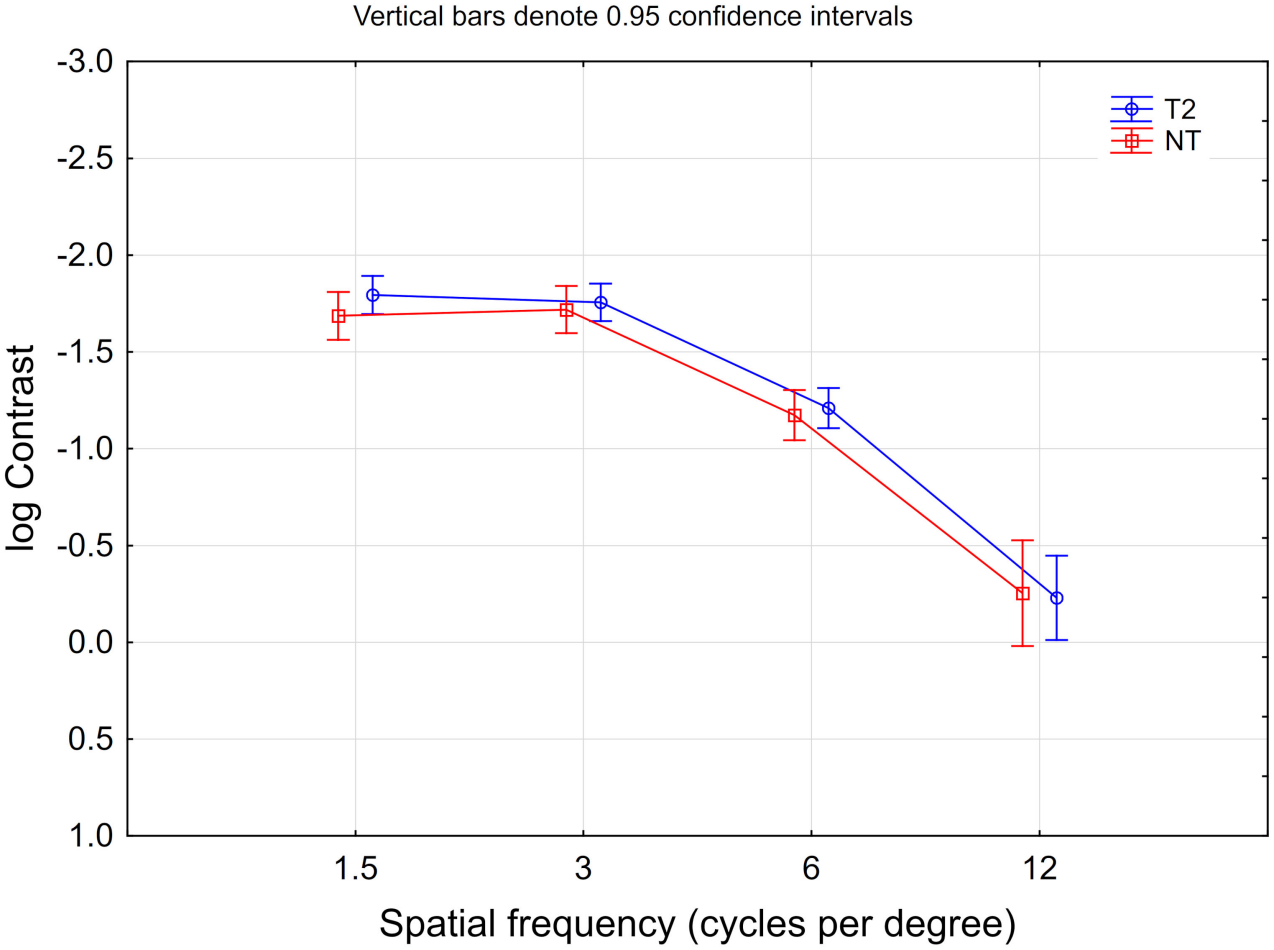
Mesopic contrast sensitivity by lens type.

The results of the contrast sensitivity testing are shown in **Figure 3** (photopic) and **Figure 4** (mesopic). There was no statistically significant difference overall between the lens groups in either the photopic (p = 0.71) or mesopic (p = 0.63) test conditions. There was also no statistically significant difference between the lens groups at any individual spatial frequency (p > 0.16 in all cases).

Lens orientation at the three-month visit was measured and compared to the planned orientation of the IOL at the time of surgery. Twenty-seven of 30 (90%) of the T2 lenses were within 5 degrees of the intended orientation. Of the three remaining lenses, two were oriented +6 degrees from intended, with residual refractive cylinder of 0.25 D in both eyes. The last lens was oriented -9 degrees from intended, with a residual refractive cylinder of 0.50 D.

One T2 lens was rotated at the 1-day visit, as it was noted to be 94 degrees from the intended axis. No other implanted lens required repositioning post-surgery. There were no adverse events, product issues or other safety events observed in this study.

A sub-analysis of the T2 results by the preoperative corneal cylinder orientation was conducted, but the low number of eyes in each subgroup necessitates caution in interpreting the results (8 eyes with-the-rule, 8 oblique and 14 against-the-rule). There was no statistically significant difference in the UDVA (p = 0.34) or the residual refractive cylinder (p = 0.70) by corneal cylinder orientation.

## Discussion

Results here support the use of a low cylinder power IOL in eyes which qualify for such a lens based on accurate biometry and a modern toric IOL calculator (including consideration of posterior corneal astigmatism). With careful orientation of the lens in the eye, patients are likely to have uncorrected distance VA a line better than can be achieved with a non-toric IOL, consistent with the expected ~ 0.50 D reduction in residual refractive cylinder. There were no negative effects of the toric IOL on distance-corrected low contrast acuity (in photopic or mesopic conditions, with or without glare) or contrast sensitivity (in photopic or mesopic conditions) noted in the current study.

There have been several previously published studies evaluating the correction of low amounts of anterior corneal astigmatism with toric IOLs. Gundersen and Potvin analyzed visual outcomes from a matched cohort of subjects implanted with an Acrysof SN6AT2 lens vs. an Acrysof SN60AT lens (both Alcon, Fort Worth, USA) using a similar test battery (11). The overall results they reported in terms of uncorrected visual acuity and low contrast acuity are close to those reported here for the Clareon lenses, though the difference in UDVA found in the current study is slightly greater. This may reflect a better match in the subject groups. Levitz et al (12), and Aujla et al (13) provided clinical outcomes for the same AcrySof T2 toric IOL, but neither included a control group; their clinical outcomes for the toric IOL were similar to those reported in the current study. Bellucci et al. reviewed a retrospective case series for eyes implanted with a 1.0 D toric IOL (PerfecTor, Hanita Lenses, Israel), comparing results to those obtained with a different monofocal IOL (Incise, Bausch & Lomb, USA) (14). The differences they observed in lens performance were lower than those reported in the previously mentioned studies, and the current study. The authors felt that this may have been due to the limitation of their retrospective study design. However, they concluded that implanting an IOL with 1.0 D of cylinder was marginally better than implanting a non-toric IOL. Hienert et al. performed a contralateral eye study with a low cylinder IOL vs. a non-toric IOL from a different manufacturer and found the same one-line difference in UDVA associated with toric correction as was reported here (15).

The nominal correction of the T2 IOL at the corneal plane is ~0.65 D. The difference in the mean residual refractive astigmatism observed in this study between the T2 and non-toric IOL was 0.36 D, somewhat lower than this. The difference between these expected and achieved results is likely due to the inherent variability in biometry, the variability in surgically induced astigmatism, and the variability in the postoperative refraction, with measurement of the preoperative corneal surface considered likely to be the most important factor (2). However, the percentage of subjects with ≤ 0.25 D and ≤ 0.50 D of residual refractive astigmatism was significantly higher in the T2 group, indicating the lens is effective.

There are limitations to the current study. Only distance VA was measured in the uncorrected state. This precluded reporting any differences in uncorrected low contrast acuity or contrast sensitivity. Evaluation of these and other visual metrics in the uncorrected state is an area for potential future research. The number of subjects enrolled was limited, precluding reliable analysis of any confounding effects, such as the effect of preoperative corneal astigmatism orientation on results. Because the eyes were evaluated monocularly, there were no measures of subjective visual quality included, as patients usually consider these binocularly.

## Conclusion

The use of a low cylinder power lens provided subjects with significantly lower residual refractive astigmatism three months after surgery, resulting in nearly one line better uncorrected distance visual acuity relative to a non-toric IOL. There were no significant differences in contrast sensitivity or low contrast acuity when measured in the distance-corrected state. The achieved cylinder correction was consistent with expectations.

## Data Availability

The raw data supporting the conclusions of this article will be made available by the authors, without undue reservation.
